# Lung Transplantation From Controlled and Uncontrolled Donation After Circulatory Death (DCD) Donors With Long Ischemic Times Managed by Simple Normothermic Ventilation and *Ex-Vivo* Lung Perfusion Assessment

**DOI:** 10.3389/ti.2023.10690

**Published:** 2023-02-08

**Authors:** Alessandro Palleschi, Alberto Zanella, Giuseppe Citerio, Valeria Musso, Lorenzo Rosso, Davide Tosi, Jacopo Fumagalli, Gianluca Bonitta, Elena Benazzi, Gianluca Lopez, Valeria Rossetti, Letizia Corinna Morlacchi, Clarissa Uslenghi, Massimo Cardillo, Francesco Blasi, Giacomo Grasselli, Franco Valenza, Mario Nosotti

**Affiliations:** ^1^ University of Milan, Milan, Italy; ^2^ Thoracic Surgery and Lung Transplantation Unit, Fondazione IRCCS Ca’ Granda - Ospedale Maggiore Policlinico, Milan, Italy; ^3^ Department of Anaesthesia, Critical Care and Emergency, Fondazione IRCCS Ca’ Granda—Ospedale Maggiore Policlinico, Milan, Italy; ^4^ School of Medicine, University of Milano - Bicocca, Milano, Italy; ^5^ Neurointensive Care, Fondazione IRCCS San Gerardo dei Tintori, Monza, Italy; ^6^ Coordinamento Trapianti North Italy Transplantation Program (NITp), Fondazione IRCCS Ca’ Granda—Ospedale Maggiore Policlinico, Milan, Italy; ^7^ Pathology Unit, Fondazione IRCCS Ca’ Granda—Ospedale Maggiore Policlinico, Milan, Italy; ^8^ Respiratory Unit and Cystic Fibrosis Adult Center, Fondazione IRCCS Ca’ Granda—Ospedale Maggiore Policlinico, Milan, Italy; ^9^ Italian National Transplantation Centre, Rome, Italy; ^10^ Department of Anaesthesia and Critical Care, Fondazione IRCCS Istituto Nazionale dei Tumori, Milan, Italy

**Keywords:** lung transplantation, chronic lung allograft dysfunction, primary graft dysfunction, donation after circulatory death donors, ischemia time, lung preservation

## Abstract

Donation after cardiac death (DCD) donors are still subject of studies. In this prospective cohort trial, we compared outcomes after lung transplantation (LT) of subjects receiving lungs from DCD donors with those of subjects receiving lungs from donation after brain death (DBD) donors (ClinicalTrial.gov: NCT02061462). Lungs from DCD donors were preserved *in-vivo* through normothermic ventilation, as per our protocol. We enrolled candidates for bilateral LT ≥14 years. Candidates for multi-organ or re-LT, donors aged ≥65 years, DCD category I or IV donors were excluded. We recorded clinical data on donors and recipients. Primary endpoint was 30-day mortality. Secondary endpoints were: duration of mechanical ventilation (MV), intensive care unit (ICU) length of stay, severe primary graft dysfunction (PGD3) and chronic lung allograft dysfunction (CLAD). 121 patients (110 DBD Group, 11 DCD Group) were enrolled. 30-day mortality and CLAD prevalence were nil in the DCD Group. DCD Group patients required longer MV (DCD Group: 2 days, DBD Group: 1 day, *p* = 0.011). ICU length of stay and PGD3 rate were higher in DCD Group but did not significantly differ. LT with DCD grafts procured with our protocols appears safe, despite prolonged ischemia times.

## Graphical Abstract

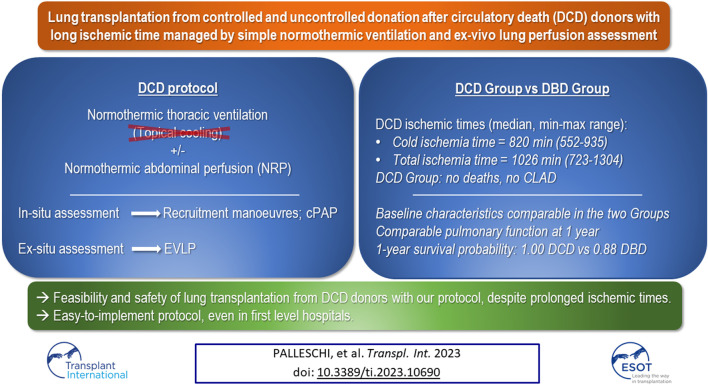

## Introduction

Lung transplantation is a well-established treatment for selected patients with end-stage benign respiratory diseases. Donor’s shortage is one of the main factors limiting lung transplantation, hence the great interest in lung procurement from donation after circulatory death (DCD) donors (1,2). Maastricht category III DCD donors are the most widely used and best studied (3). Conversely, the uncontrolled settings of categories I and II are fascinating but challenging for at least three reasons: timing, organ preservation, and assessment. On the other hand, while using category III DCD lungs avoids these issues, it gives rise to ethical concerns about the withdrawal of life-sustaining treatment (WLST). In this scenario, few lung transplantation centres established an uncontrolled DCD (uDCD) program, and even fewer have protocols including both controlled (cDCD) and uncontrolled DCD settings (4).

The impact of the legal and ethical system of the different countries is relevant. In Italy, DCD is legal but suffers from the 20 min of recorded flat electrocardiogram (EKG) required for death declaration. WLST is also allowed by Italian law but has not become common practice yet and has been only recently codified. After a long pre-clinical phase (5,6,7), we refined an original two-steps protocol to manage lungs from DCD donors to overcome the long acirculatory period (8). First, by leveraging the possibility of dissociating ischemia from hypoxia, we adopted an open lung strategy for *in-situ* lung preservation even for prolonged periods, without topical cooling. In the second phase of the protocol, we employed the *ex-vivo* lung perfusion (EVLP) for *ex-situ* graft evaluation and reconditioning. We began our experience with the uncontrolled setting in 2014, and then followed the same principles when dealing with the controlled one (9).

Here we present the results of a clinical trial comparing the outcomes after lung transplantation of subjects receiving lungs from DCD donors with those of subjects who received lungs procured from donation after brain death (DBD) donors.

## Patients and Methods

### Study Design

This study is a single-institution, prospective cohort trial (ClinicalTrial.gov: NCT02061462). We wanted to verify the safety of lung transplantations performed with organs from DCD donors procured with an original protocol. We compared clinical and functional outcomes of patients undergoing lung transplantation who received grafts from DCD donors (DCD Group) with those of recipients of lungs from DBD donors (DBD Group) in our centre in the same period. We also performed an analysis comparing the outcomes of the DCD Group with those of recipients of lungs from DBD donors requiring machine perfusion (DBD-EVLP Group).

Since November 2014 all subjects provided written informed consent to participate in the trial at the time of enlisting, in accordance with the protocol approved by the local Ethics Committee. Recipients were selected sequentially, based on blood group, size match (total lung capacity) and waiting-list status (i.e., lung allocation score (LAS) or emergency program) (10,11). The type of donor (DBD vs. DCD) did not represent a criterion for donor-recipient matching, thus maintaining randomness of recipients group distribution. Recipients of a DCD lung were asked to renew their consent to receive organs from a DCD donor closely ahead of transplantation. During and after surgery, standard care was provided in both groups, according to our protocol (see Supplementary Material). Recipients’ and respective donors’ variables of interest were recorded in a dedicated electronic database from the date of waiting-list entry to the date of the last follow-up. Institutional board approval for data use was obtained (number 749_2016bis). The follow-up period was concluded on 31st July 2020.

### Inclusion and Exclusion Criteria

All patients enlisted for bilateral lung transplantation older than 14 years were deemed eligible. Candidates for multi-organ transplantation or re-transplantation were excluded. Donors aged 65 and older, as well as DCD category I or IV donors, were also excluded.

### Lung Allocation Process and Procurement Protocol

The lungs, from both DBD and DCD donors, were offered to our centre by the regional and national organ procurement organizations: the North Italian Transplant program (NITp) and the National Transplantation Centre (CNT), respectively (10,12). Notably, Italian law requires 6 h of observation, or 20 min of flat EKG to declare the patient’s death according to neurological or cardiocirculatory criteria, respectively.

#### Donor Selection Criteria

Both DBD and DCD donor lungs suitability was determined according to standard criteria. Donors with massive lung contusions, history of aspiration of gastric content, pneumonia, or sepsis were excluded. Regarding DCD Maastricht type II donors, subjects with cardiovascular collapse, first treated by an advanced life support crew on the scene, then transferred to the emergency room, were considered as potential donors if declared dead after advanced cardiac life support attempts had failed (8). DCD Maastricht type III were patients admitted to the intensive care unit (ICU), where cardiac arrest occurred after a planned WLST. The following were considered DCD donors refusal criteria: unwitnessed cardiac arrest; no-flow (preceding initiation of cardio-pulmonary resuscitation, CPR) period >15 min and/or low flow >60 min for uDCD; *in-situ* warm ischemia time (WIT) >240 min for both cDCD and uDCD.

#### DBD Preservation and Procurement Protocol

Our lung procurement procedure consists of a standard bi-pulmonary block retrieval Once organs have been prepared for retrieval and the pulmonary artery (PA) is cannulated, prostaglandin E1 (500 mcg) is injected into the PA, aortic and venae cavae cross-clamp is performed, the left atrial appendage is amputated and/or the posterior aspect of the left atrium is incised, and the anterograde pulmonary flush is performed with 60 mL/kg of cold (4°C–8°C) PerfadexTM. The retrograde pulmonary flush is performed by using 250 mL PerfadexTM per pulmonary vein.

#### DCD Preservation and Procurement Protocol

Our protocol for lung preservation and retrieval has been previously described in detail (8,9). In short, it consists of a non-rapid normothermic open-lung procurement, namely without pleural topical cooling (i.e., without chest tube placement) before the start of cold flushing. In uDCD donor’s management, after heart beating cessation, 5 min of no-touch period are required to clinically confirm the diagnosis of death. A recruitment manoeuvre (RM) is performed by progressively increasing airway pressure over a positive end-expiratory pressure (PEEP) of 5 cmH_2_O to obtain a total airway pressure of 35 cmH_2_O with 10 bpm of respiratory rate and inspiratory/expiratory [I/E] ratio 1:1. Continuous positive airways pressure (CPAP 10 cmH_2_O, 100% FiO_2_) is applied until death is confirmed according to circulatory criteria (20 min of flat EKG). Heparin is given (10.000 IU by endovenous push, followed by 3 min of CPR), a new RM is performed, and ventilation is started (respiratory rate 4 breaths/min, tidal volume 6 mL/kg, PEEP 8 cmH_2_O, fraction of inspired oxygen [FiO_2_] 100%, I/E ratio 1:1). If chest radiographs and bronchoscopic evaluation are normal, the subject is transferred to the operating room, and lung procurement is performed. Lungs are perfused *in situ* with a fibrinolytic agent (15 mg of recombinant tissue plasminogen activator, rTPA), flushed with Perfadex^TM^ (60 mL/kg anterograde) and procured (see Supplementary Material for further details).

Similarly, in case of cDCD, after re-intubation of the donor, lung preservation is achieved through protective mechanical ventilation (13). If combined procurement with abdominal organs is proposed, we associate a non-rapid normothermic open-lung strategy with the abdominal normothermic regional perfusion (NRP), as described in the Supplementary Material (9).

#### *Ex-Vivo* Lung Perfusion

We utilize a custom-made circuit to perform EVLP procedures according to a protocol previously described (14). For the purpose of this trial, EVLP has been used in the following cases:

- lungs from DBD donors with PaO_2_/FiO_2_ <300 mmHg on a PEEP of 5 cmH_2_O and/or with chest X-ray abnormalities after optimization of MV
- lungs from DBD donors on veno-arterial extracorporeal membrane oxygenation (ECMO) for cardio-circulatory support during brain death observation, whose evaluation of gas exchange is suboptimal
- lungs from DCD donors.

At the end of the EVLP protocol, lungs were judged suitable for transplantation according to criteria described elsewhere (15).

#### Ischemia Times Definition

- Cold ischemia time (CIT): the time between lung cold flushing and beginning of organ implantation (without considering machine perfusion time) for both DBD and DCD grafts.
- Intraoperative WIT: the time from the beginning of organ implantation to reperfusion.
- Total ischemia time (TIT): the time from cross-clamping to reperfusion for the DBD group; from cardiac arrest to reperfusion for category II DCD donors; from the drop of systolic blood pressure <50 mmHg to reperfusion for category III DCD donors. TIT did not include machine perfusion time.
- Total preservation time (TPT): the time from cross-clamping to reperfusion for DBD donors, from the end of CPR to reperfusion for category II DCD donors, and from cardiac arrest to reperfusion for category III DCD donors. TPT included machine perfusion time.

Finally, for category II DCD donors, we also considered a WIT period from cardiac arrest to pulmonary cold flush; for category III DCD donors, we recorded interval 1, 2, 3 and 4 as suggested by the International Society for Heart and Lung Transplantation (ISHLT) (16). For more details on the procurement process see Supplementary Material.

### Study Endpoints

Primary endpoint was the 30-day mortality after transplantation. The secondary endpoints were the duration of MV, ICU length of stay, the occurrence of primary graft dysfunction (PGD) of grade 3 within the first 72 h after transplantation (17), and the onset of chronic lung allograft dysfunction (CLAD) (18).

### Statistical Analysis

Continuous data are presented as median and inter quartile range (IQR). Binary variables are shown as absolute and percentages frequencies. The Mann-Whitney or Chi-square tests were performed, as appropriate. Pulmonary function parameters were measured at 3, 6, and 12 months. The repeated measures for pulmonary function data were analysed using the “mean response profile” method through generalized estimating equations (GEE) by employing time as a categorical variable and logit link function (19). GEE standard errors were calculated with a sandwich estimator. We used the unstructured working correlation matrix selected by correlation information criterion (20). The GEE regression model was adjusted by donor smoking history, donor age, donor-recipient sex mismatch, surgical incision, recipient medical diagnosis, LAS, grade 3 PGD, TIT and WIT for both first and second lung, airway complications.

We chose GEE because it allows a population-averaged interpretation of the regression coefficients. The null hypothesis was that the difference of pulmonary function between the two study groups was constant over time. This was verified using the multivariate Wald test, testing time × group interaction in the GEE regression model. Profile likelihood confidence intervals (CIs) at 95% confidence level were computed. Univariate Wald test for each GEE-estimated parameter was performed. The non-parametric Kaplan-Meier estimator was used to analyse time-to-event data related to overall survival and CLAD onset. Confidence intervals (CIs) were at 95% and 2-sided *p*-values were calculated. A *p*-value of <0.05 was considered statistically significant; the inference should be intended for exploratory purposes. All analyses and graphs were carried out using an R software (version 3.2.2) (21).

## Results

### Study Population

**FIGURE 1:**
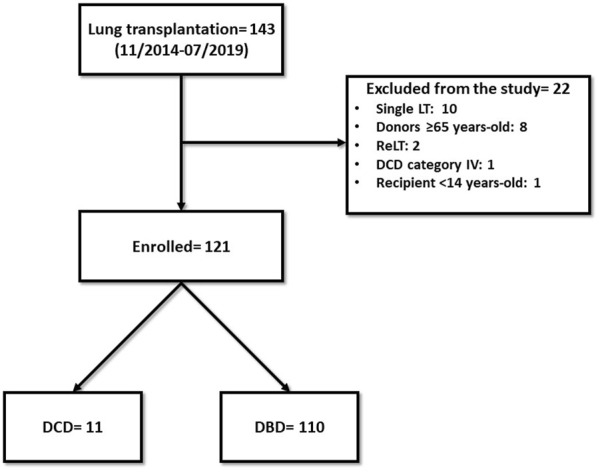
Study population flow-chart. LT: lung transplantation; ReLT: retransplantation.

**FIGURE 2:**
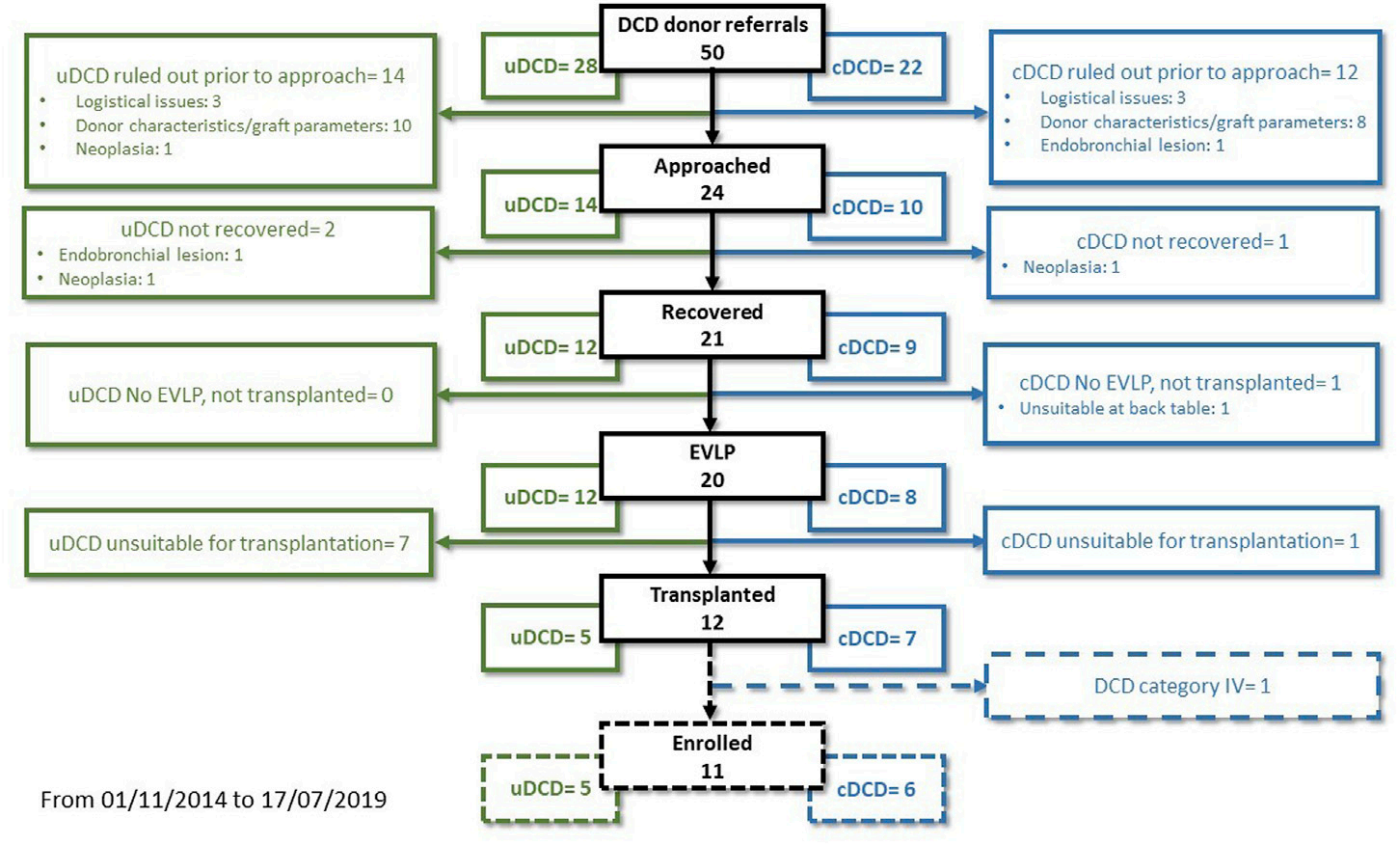
Overall flow-chart of our DCD program.

From November 2014 to July 2019, we performed 143 lung transplantations. Out of these, 22 cases were excluded from the study: ten single-lung transplantations, eight donors ≥65 years-old, two re-transplantations, one DCD category IV donor, one recipient <14 years-old (Figure 1). The remaining 121 patients were enrolled in the study: 110 in the DBD Group and 11 in the DCD Group (five patients received lungs procured from DCD II donors and six from DCD III donors). The complete results of our DCD program are shown in Figure 2.

Recipients’ diseases leading to lung transplantation were distributed homogeneously in the two groups; notably, in the DCD and DBD groups, 72.7% and 61.9% of patients suffered from cystic fibrosis (CF), respectively. Recipients were similar in terms of number of urgent transplants, LAS, preoperative mean pulmonary artery pressure (PAPm), and preoperative arterial partial pressure of carbon dioxide (PaCO_2_). Sex mismatch (female recipient with male donor) occurred more often in the DCD Group (*p* = 0.009) (Table 1). There were no statistically significant differences between the DBD and DCD groups in terms of donors’ sex, age, comorbidities, duration of MV, smoking history, percentage of abnormal chest X-ray, PaO_2_/FiO_2_ ratio, and secretions at bronchoscopy. Donor BMI was significantly higher in the DCD Group (*p* = 0.022). The percentage of grafts from DBD undergoing machine perfusion was 15.5%; all grafts from DCD donors underwent *ex-vivo* evaluation. With regard to perioperative variables, the two groups were similar in terms of type of incision, need for both intraoperative and post-operative extra-corporeal support, and packed red blood cell, plasma and platelet intraoperative transfusion.

**TABLE 1:** Study population demographic and clinical characteristics.

	DCD Group (*n* = 11)	DBD Group (*n* = 110)	*p*-value
Donor characteristics			
Sex (Female), n (%)	1 (9.1)	41 (37.3)	0.061
Age, median (IQR)	53.0 (8.5)	49.0 (19.0)	0.168
BMI, median (IQR)	27.7 (5.5)	24.8 (4.4)	0.022^a^
Comorbidities (Yes), n (%)	7 (63.6)	48 (43.6)	0.204
Mechanical ventilation (days), median (IQR)	1 (6)	2 (3)	0.611
Smoking history, n (%)			
No	7 (63.6)	73 (66.3)	0.855
Former	1 (9.1)	8 (7.3)	0.827
Yes	3 (27.3)	29 (26.4)	0.948
Chest X-ray, n (%)			
Clear	7 (63.6)	69 (62.7)	0.952
Minor	1 (9.1)	25 (22.7)	0.294
Opacity<1 lobe	3 (27.3)	12 (10.9)	0.116
Opacity ≥1 lobe	0 (0.0)	4 (3.7)	0.520
Donor PaO_2_/FiO_2_, median (IQR)^b^	372.0 (60.0)	470.0 (137.0)	0.07
Donor secretions, n (%)			
None	5 (45.4)	43 (39.1)	0.681
Minor	3 (27.3)	53 (48.2)	0.185
Moderate	3 (27.3)	13 (11.8)	0.149
Major	0 (0.0)	1 (0.9)	0.751
Sex mismatch, n (%)	5 (45.5)	16 (14.5)	0.009*
Recipient characteristics			
Sex (Female), n (%)	6 (54.5)	52 (47.3)	0.645
Age, median (IQR)	42.0 (17.5)	36.5 (24.8)	0.836
BMI, median (IQR)	20.8 (3.2)	20.5 (4.2)	0.960
Disease, n (%)			
Cystic fibrosis	8 (72.7)	68 (61.9)	0.475
Interstitial lung disease	1 (9.1)	25 (22.7)	0.294
COPD	2 (18.2)	10 (9.1)	0.336
Bronchiectasis	0 (0.0)	3 (2.7)	0.579
Pulmonary vascular disease	0 (0.0)	1 (0.9)	0.751
Other	0 (0.0)	3 (2.7)	0.579
Urgent transplantation, n (%)	0 (0.0)	10 (9.1)	0.297
LAS, median (IQR)	42.9 (9.0)	38.5 (12.6)	0.652
PAPm, median (IQR)	22.0 (2.0)	24.0 (10.0)	0.924
PaCO_2_, median (IQR)	48 (8)	42 (11)	0.362
ECMO Bridge to transplantation, n (%)	0 (0.0)	10 (9.1)	0.297
Intraoperative			
Incision, n (%)			
Clamshell	9 (81.8)	71 (64.5)	0.249
Bilateral anterior thoracotomy	2 (18.9)	39 (35.5)	0.249
Machine perfusion, n (%)	11 (100)	17 (15.5)	<0.001^a^
CIT, 1st lung (minutes), median (IQR)	595 (159)	338 (157)	<0.001^a^
CIT, 2nd lung (minutes), median (IQR)	820 (155)	557 (181)	<0.001^a^
Intraoperative WIT, 1st lung (minutes), median (IQR)	76 (27)	82 (29)	0.257
Intraoperative WIT, 2nd lung (minutes), median (IQR)	80 (24)	71 (25)	0.539
TIT, 1st lung (minutes), median (IQR)	797 (154)	428 (156)	<0.001^a^
TIT, 2nd lung (minutes), median (IQR)	1,026 (202)	632 (186)	<0.001^a^
TPT, 1st lung (minutes), median (IQR)	1,058 (125)	433 (175)	<0.001^a^
TPT, 2nd lung (minutes), median (IQR)	1,286 (102)	641 (213)	<0.001^a^
ECMO, n (%)	7 (63.6)	51 (46.4)	0.274
Intraoperative Red cells concentrate transfusion (U), median (IQR)	3.0 (4)	3.0 (5)	0.899
Intraoperative Plasma transfusion (U), median (IQR)	1.0 (3)	0.0 (3)	0.778
Intraoperative Platelet transfusion (U), median (IQR)	0.0 (0)	0.0 (0)	0.396

^a^ Statistically significant *p*-value.

^b^ Median PaO_2_/FiO_2_ was calculated in 5 and 106 patients in the DCD and DBD Group, respectively.

BMI, body mass index; IQR, interquartile range; PaO_2_/FiO_2_, ratio of arterial oxygen partial pressure (PaO_2_ in mmHg) to fractional inspired oxygen; COPD, chronic obstructive pulmonary disease; LAS, lung allocation score; PAPm, mean pulmonary artery pressure; PaCO_2_, arterial partial pressure of carbon dioxide; ECMO, extracorporeal membrane oxygenation; CIT, cold ischemia time; WIT, warm ischemia time; TIT, total ischemia time; TPT, total preservation time; U, units.

CIT, TIT, and preservation times were significantly higher in the DCD Group (*p* < 0.001), while intraoperative WIT was similar between the two groups. Table 2 shows the ischemia times of procurement and preservation in the DCD Group, in details.

**TABLE 2:** DCD Group ischemic times (16).

DCD category III (*n* = 6)	
Interval 1 (minutes), mean (SD)	14.3 (8.5)
Interval 2 (minutes), mean (SD)	26 (8.9)
Interval 3 (minutes), mean (SD)	165.5 (29.9)
Interval 4 (minutes), mean (SD)	151 (37)
DCD category II (*n* = 5)	
WIT (minutes), mean (DS)	250 (53)

Interval 1: from WLST to BP<50mmHg; Interval 2: WLST to asystole; Interval 3: WLST to pulmonary flushing; Interval 4: BP<50mmHg to pulmonary flushing. SD: standard deviation.

### Post-Operative Course and Outcomes

Post-operative data and outcomes are shown in Table 3. No adverse events related to our protocol were recorded in the DCD Group. In the first 30 days 1 patient (0.9%) died in the DBD Group, none in the DCD Group (p = NS). There was a statistically significant difference in median duration of MV (2 days for DCD and 1 day for DBD, *p* = 0.011). The prevalence of PGD3 within the first 72 h was 27.3% in the DCD Group and 18.2% in the DBD Group (*p* = 0.742).

**TABLE 3:** Patients’ postoperative data and outcomes.

	DCD Group (*n* = 11)	DBD Group (*n* = 110)	*p*-value
Grade 3 PGD, n (%)	3 (27.3)	20 (18.2)	0.724
MV (days), median (IQR)	2.0 (2.5)	1.0 (2.0)	0.011*
ICU stay (days), median (IQR)	4.0 (5.5)	3.0 (3.0)	0.053
Hospital stay (days), median (IQR)	21 (5)	22 (10)	0.732
Airway complication, n (%)	2 (18.2)	7 (6.4)	0.154
90-days mortality	0 (0.0)	5 (4.6)	0.999
ALAD, n (%)	3 (27.3)	34 (30.9)	0.999
Histologic AR^a^, n (%)			
Grade 0	4 (36.3)	28 (28)	0.561
Grade 1	5 (45.5)	56 (56)	0.505
Grade 2	2 (18.2)	14 (14)	0.708
Grade 3	0 (0.0)	2 (2)	0.636

^a^ AR was calculated in 11 DCD and 100 DBD.

IQR, interquartile range; PGD, primary graft dysfunction; MV, mechanical ventilation; ICU, intensive care unit; ALAD, acute lung allograft dysfunction; AR, acute rejection.

Airway complications occurred in two recipients of DCD Group (18.2%) and seven of the DBD Group (6.4%): the difference, however, did not reach statistical significance (*p* = 0.154). No bronchial anastomotic dehiscence occurred in both groups, but only stenosis. Both cases and the 71.4% of patients required endoscopic treatment in the DCD Group and DBD Group, respectively.

**FIGURE 3:**
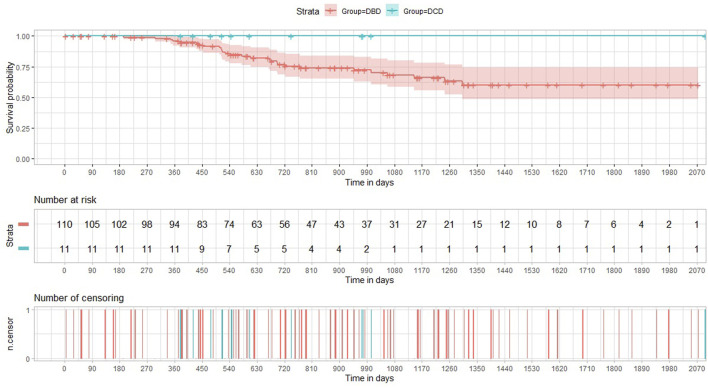
Kaplan-Meier curves illustrating CLAD-free survival in the DCD and DBD Group.

**FIGURE 4:**
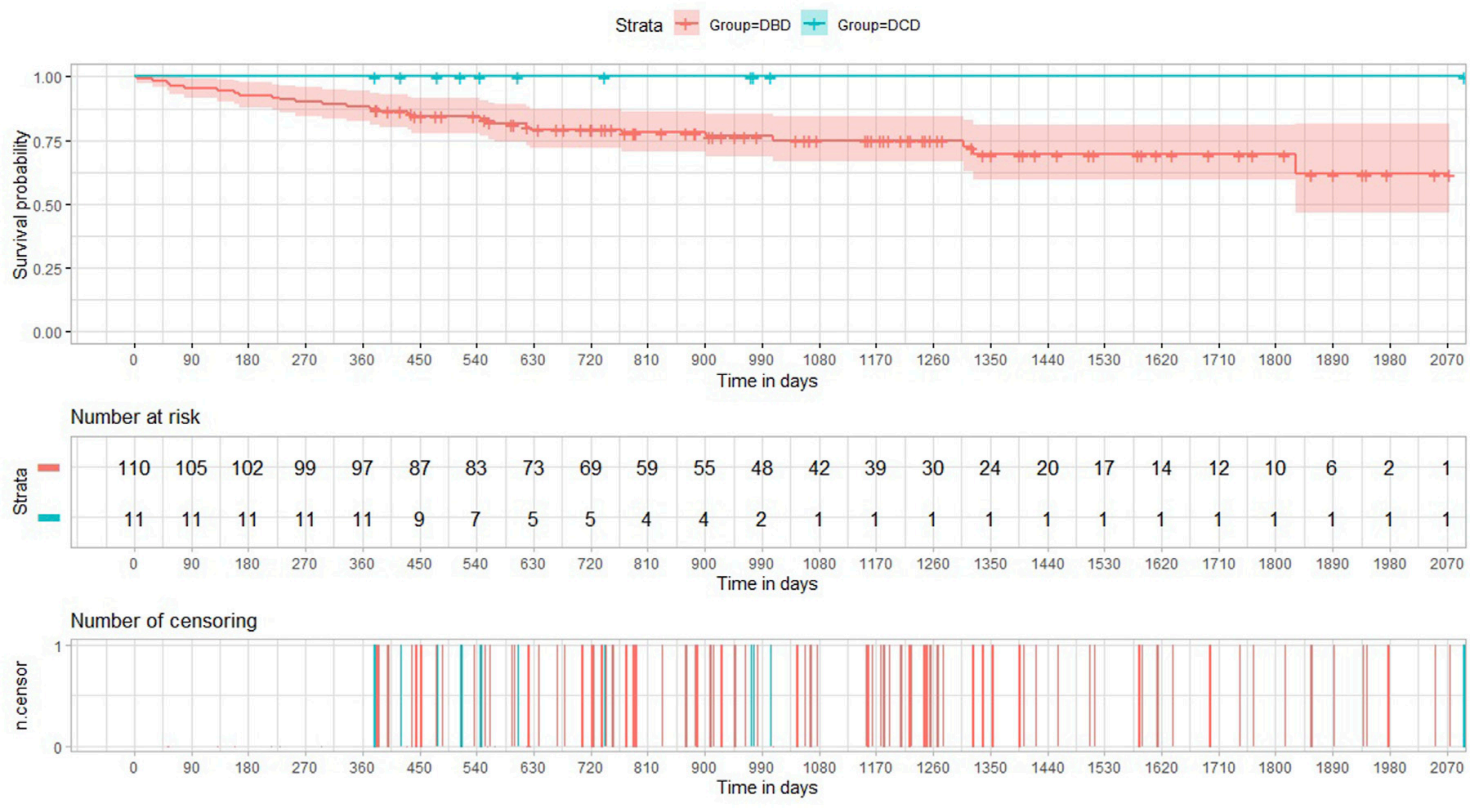
Kaplan-Meier curves illustrating overall survival in the DCD and DBD Group.

The incidence of both histology-proven acute rejection (AR) and acute lung allograft dysfunction (ALAD) was similar between the two groups (22). The median follow-up period after transplantation was 605 days in the DCD Group and 895 days in the DBD Group. None of the patients receiving lungs from a circulatory death donor experienced CLAD during the period of the study. The probability of CLAD free survival in the DBD Group at 1, 3 and 5 years after transplantation was 0.96, 0.68, and 0.60, respectively (Figure 3). There were no deaths in the DCD Group, while overall survival in the DBD Group at 1, 3 and 5 years after surgery was 0.88, 0.75 and 0.70, respectively (Figure 4).

### Pulmonary Function

**FIGURE 5:**
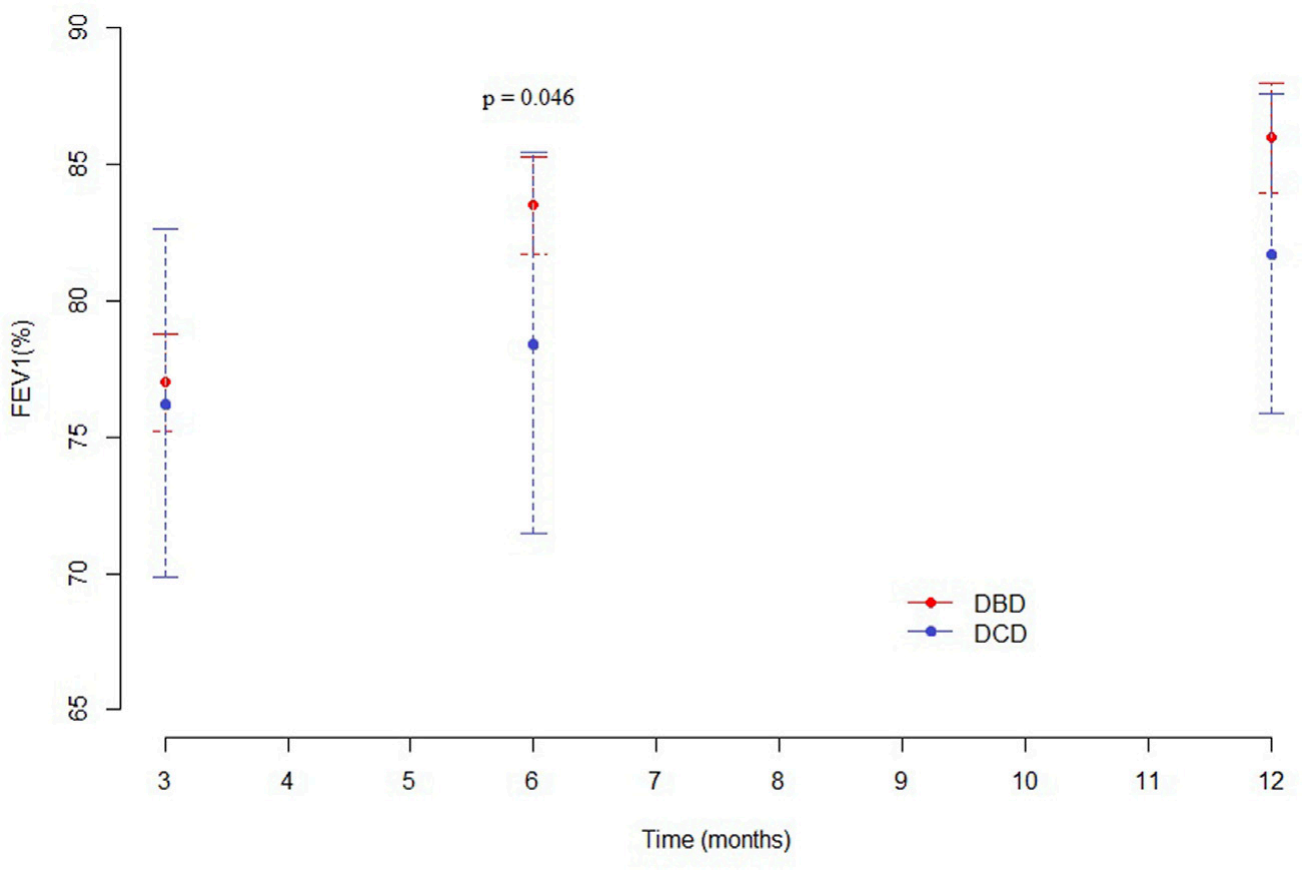
Mean FEV1 (%) at 3, 6, and 12 months after transplantation.

**FIGURE 6:**
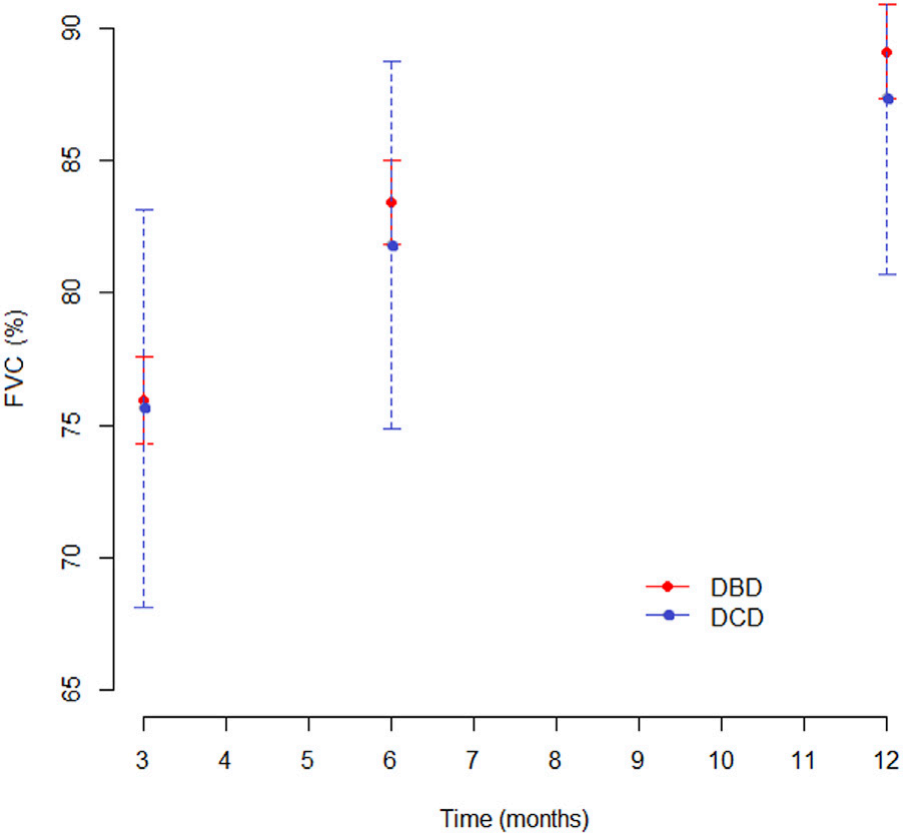
Mean FVC (%) at 3, 6, and 12 months after transplantation.

At 3 months, pulmonary function values were similar in both groups for Forced Expiratory Volume in the first second (FEV1), forced vital capacity (FVC) and Tiffeneau index. Mean percentage of predicted FEV1 at 3, 6 and 12 months was 76.3%, 78.5%, and 81.7% in the DCD Group and 77%, 83.5%, and 86% in the DBD Group, respectively (Supplementary Table S1). The difference in mean FEV1 was statistically significant only at 6 months (*p* = 0.046), as shown in Figure 5. Mean FVC and Tiffeneau index were not significantly different at all time points (Figure 6).

The results of the adjusted GEE regression analysis for pulmonary function tests cohort are shown in Supplementary Table S2. At the test baseline (3 months), lower FEV1 and FVC were associated with grade 3 PGD and clamshell incision. Moreover, grade 3 PGD, donor age, and airway complications significantly reduced the Tiffeneau index.

Data regarding best FEV1 reached within the first year after transplantation and throughout the follow-up period are shown in Table 4. There were no statistically significant differences between the two groups. In both sets of patients, best FEV1 was reached during the first year after surgery: in the DCD Group, the median time to achieve best FEV1 was 217 days, whereas in the DBD Group, it was 333 days.

**TABLE 4:** Best FEV1 values in the DCD and DBD Groups.

	DCD Group	DBD Group	*p*-value
Best FEV1 within 1st year (%), median (IQR)^a^	97 (38)	87 (23.8)	0.7937
Time to reach best FEV1 within 1st year (days), median (IQR)^a^	196 (149)	234 (184)	0.3297
Best FEV1 (%), median (IQR)	99 (33)	90 (25)	0.7107
Time to reach best FEV1 (days), median (IQR)	217 (486)	333 (455)	0.9129

^a^ Best FEV1 within the first year and days for reaching best FEV1 within the first year were calculated in 11 DCD and 105 DBD.

FEV1, forced expiratory volume in the first second; IQR, interquartile range.

### DCD Group vs. DBD-EVLP Group

When comparing the DCD Group with the subset of DBD grafts undergoing EVLP, the difference in terms of duration of MV did not reach statistical significance. The incidence of PGD3 in the DBD-EVLP Group was 23.5% vs. 27.3% in the DCD Group. Also, there was no statistically significant difference regarding airway complications, even though the incidence was higher in the DCD Group (18.2% vs. 5.8% in the DBD-EVLP Group).

The punctual estimates and regression as a function of time of FEV1, FVC, and Tiffeneau index are presented for the DCD Group and DBD-EVLP Group in Supplementary Table S3 in the Supplementary material. No difference was detected between the two groups.

## Discussion

We present the results of a prospective trial designed to compare the outcomes of patients receiving grafts from DBD and DCD donors, managed with our original protocol. Our experience suggests that transplantation from both controlled and uncontrolled DCD donors is feasible and safe even after prolonged ischemic times in a non-rapid procurement setting. Notably, the protocol has been activated in first level as well as in secondary hospitals (4,8,9,12,23,24).

In Italy, a mixed “opting-in” and “opting-out” system and, more importantly, 20 min of flat EKG for the declaration of circulatory death were long considered an insurmountable obstacle to the use of DCD donors. Our protocol relied on the possibility for lung tissue to dissociate ischaemia from hypoxia, hence the preservation of lungs for an extended time by using RMs followed by continuous positive airway pressure (*in-situ* preservation phase), and subsequently evaluating them by using EVLP (*ex-situ* preservation phase)(8). Our peculiar strategy allowed a complete expansion of the lungs for optimal perfusion, instead of causing a parenchymal collapse for topical cooling (25). The advantages of this approach were recently confirmed by the work of Healey et al. (26). Also, employing only ventilation allowed us to preserve the lung without the need for chest drains and topical cooling, making it possible to implement our protocol in any situation, also in first level hospitals. Finally, we combined this approach with the NRP for abdominal organ preservation (9,13). Indeed, the ischemia times of our DCD cohort were generally longer than those reported in the literature. Our median TIT was three times greater than that reported by Cambridge or Harefield Center (27,28). The analysis of the mean WIT in DCD category II donors shows that prolonged *in-situ* WIT was not strictly considered a refusal criterion, as we prefer to evaluate the grafts on a case-by-case basis. The acceptance rate in our DCD program was much higher in category III DCD donors than in category II (0.78 versus 0.42) (Figure 2).

PGD3 rate was slightly higher in the DCD Group, although the difference did not reach statistical significance; moreover, the duration of MV was significantly longer in the DCD Group, even though the difference with DBD group was only 24 h. Finally, it is interesting to note that the PGD3 rate and pulmonary function at 1 year in the DCD Group were similar to those found in the subgroup of patients belonging to the DBD Group who received lungs treated with EVLP. Overall, DCD lungs seem to have a slower recovery in the early post-operative period.

We did not register any immediate bronchial anastomosis complications that could endanger patients’ life; in contrast, two patients in the DCD Group developed bronchial stenosis, distally to the anastomosis, in the medium term. Given the small population in the DCD cohort, the complication rate rose to 0.18. This rate is however consistent with the literature (range: 0.05–0.28) and, above all, our patients required only endoscopic treatments. Anyway, we can speculate that the length of ischemia times played a role in this regard, and that our protocol based on ventilation is more protective on pulmonary parenchyma than on large bronchi.

The medium-term outcomes of DCD lungs are encouraging. In the first year we found a homogeneous distribution of acute rejection and ALAD episodes in the two groups. To the best of our knowledge, we are the first to report the prevalence of ALAD in a population of patients transplanted with lungs by DCD donors. Sabashnikov describes acute rejection rates similar to ours and well balanced between DCD and DBD groups (28). It is possible to speculate that grafts from DCD donors have no particular impact on immunity, and therefore on the onset of rejection. One-year survival probability in the DCD Group was 1.0 versus 0.88 in the DBD Group. This finding is congruent with the result of our recent meta-analysis, where the odds ratio for 1-year overall survival was balanced between the DCD and DBD group (29). Finally, in the DCD Group, no CLAD events were recorded during the study period. Obviously, these two last results should be taken with caution considering the small sample size of our study.

The DCD Group pulmonary function was also adequate. We noted that the FEV1 was similar in the two groups 3 months after transplantation, while the recovery in the following 3 months was faster in the DBD Group: as a consequence, we observed a statistically significant difference at 6 months. One year after transplantation, this difference was no longer detectable. Despite the statistical significance reached at the six-month, it should be pointed out that such a slight difference in percentage FEV1 is clinically irrelevant. Data on Best FEV1 did not reveal any statistically significant difference between the two Groups.

This trial has several limitations. Although it is a phase I-II trial, the study population should be larger. Nevertheless, we considered it useful to perform the analyses so as not to dilute cases over time and not to expose the data set to subsequent revisions due to the continuous evolution of knowledge in the lung transplantation field. A practical example is CLAD, of which the definition is constantly evolving. We did not register CLAD cases in the study cohort, but we preferred not to comment this result in light of the absence of a clear and definitive classification (30). Another important limitation is the lack of randomization of recipients; on the other hand, the allocation system makes randomization virtually impossible. It should be noted that our two cohorts of recipients had completely overlapping parameters. Moreover, this study suffers from the possibility of generalization, being based on an original procurement protocol. Despite all this, our study can help to broaden the general knowledge on donation from circulatory arrest, which represents a possible tool to fight the chronic scarcity of lungs. Finally, we have included both category II and category III donors in the DCD group. This could be considered a confusing factor, but the examination of the two subgroups did not reveal differences in outcomes, while their merging gave an overall picture of the results obtained with our procurement protocol.

The results of this trial suggest that lung transplantation with grafts from DCD donors procured with our protocols is safe despite the long ischemia times. The trend towards a higher PGD3 rate, prolonged mechanical post-operative ventilation, and a slower functional recovery after transplantation does not seem to negatively affect clinical outcomes nor pulmonary function at 1 year.

## Data Availability

The raw data supporting the conclusion of this article will be made available by the authors upon reasonable request.

## Abbreviations

ALAD, acute lung allograft dysfunction; AR, acute rejection; cDCD, controlled donation after cardiac death; CF, cystic fibrosis; CIT, cold ischemia time; CLAD, chronic lung allograft dysfunction; CPAP, continuous positive airway pressure; CPR, cardio-pulmonary resuscitation; DBD, donation after brain death; DCD, donation after cardiac death; ECMO, extracorporeal membrane oxygenation; EKG, electrocardiogram; EVLP, *ex-vivo* lung perfusion; FEV1, Forced Expiratory Volume in the first second; FVC, forced vital capacity; GEE, generalized estimating equations; ICU, intensive care unit; LAS, Lung Allocation Score; MV, mechanical ventilation; NRP, normothermic regional perfusion; PaCO_2_, arterial partial pressure of carbon dioxide; PAPm, mean pulmonary artery pressure; PEEP, positive end-expiratory pressure; PGD, primary graft dysfunction; RM, recruitment manoeuvre; TIT, total ischemia time; TPT, total preservation time; uDCD, uncontrolled donation after cardiac death; WIT, warm ischemia time; WLST, withdrawal of life-sustaining treatment.
