# Supplementary material for: Lung Transplantation From Controlled and Uncontrolled Donation After Circulatory Death (DCD) Donors With Long Ischemic Times Managed by Simple Normothermic Ventilation and Ex-Vivo Lung Perfusion Assessment
Source: Transpl Int. 2023 Feb 8;36:10690. doi: 10.3389/ti.2023.10690 (PMC9945516; doi:10.3389/ti.2023.10690)
Supplement: Supplementary file 1 [file DataSheet1.docx]

**Supplementary material**

**Procurement protocol**

In Italy, a soft opting-out system for organ donation is currently in place: there is a presumption in favour of donation if the prospective donor has not opted out. Nevertheless, families can oppose the donation and are given the final say. Potential donors are usually identified by intensivist or emergency room staff, who refer them to the regional coordinating centre. Unless there is an urgent patient on the national urgent waiting list, the lungs are allocated within the region. In our region, organs have been assigned using LAS since 2016. Identification of potential DBD or DCD donors, decisions on treatment or CPR withdrawal, management of the dying process, and determination of death were strictly separated from the donation/transplant process. The procurement team oversaw donor management after death was declared by a local transplant coordinator.

**Normothermic regional perfusion (NRP)**

If combined procurement with abdominal organs is proposed, we associate a non-rapid normothermic open-lung strategy with the abdominal normothermic regional perfusion (NRP). Abdominal NRP is established percutaneously through the femoral vessels; a Fogarty balloon is inflated in the supraceliac aorta to exclude head and thorax. Meanwhile, bronchoscopy is performed. A first cycle of RMs is completed, and ventilation starts in a protective setting (6 ml/kg ideal body weight, FiO_2_ = 100%). A median sternotomy is then performed, and the pericardium is opened; the inferior vena cava (IVC) is encircled and closed with a tourniquet at the cavo-atrial junction, after the withdrawal of the tip of the NRP venous cannula below the level of the diaphragm. The pleurae are opened wide to allow a meticulous parenchymal inspection and to complete the suction of any existing effusion. A second RM, together with careful manipulation, gradually allows for complete lungs expansion under direct vision. Protective ventilation is resumed. The superior vena cava is ligated right below the azygos vein take-off, and the ascending aorta is clamped. The first NRP test is performed while monitoring the venous return, pump flow rate and lactate trend. In the case of a not amendable modification of the NRP flow, the lung procurement is suspended, and the tourniquet is loosened. If no detrimental change occurs, the main PA is cannulated; prostaglandin E1 is injected directly in the PA with a subsequent heart massage. Anterograde cold flush with a low-potassium dextran solution (Perfadex^TM^) is started; rTPA is added to the first bag. The tips of left and right appendages are amputated to allow the free drainage of the solution, and the posterior aspect of the left atrium is incised. At this time, cold saline solution is used for topical cooling of both lungs. After flushing is completed, the second abdominal NRP test is performed at pump flow rate; if successful, the IVC is clamped definitively; usual cardiectomy and *in situ* retrograde flushing are completed (250 ml/pulmonary vein). After retrieval, the lung block is stored on ice, and meticulous haemostasis of the chest is ensured. NRP is continued as long as necessary for abdominal organ preservation, specifically for at least 4 hours.

**EVLP**

The circuit consists of a blood reservoir connected to a gas oxygenator with a built-in heat exchanger, a centrifugal pump, a cloth arterial filter, and 0.375-inch heparin-coated polyvinyl tubing. The system is primed with 2000 ml of Steen solution (Vitrolife, Gothenburg, Sweden), 1 g of methylprednisolone, 20.000 IU of heparin and antibiotic according to donor’s and recipient’s characteristics. Two recipient-compatible packed red blood cell bags are added to the perfusate, targeting a haematocrit of 10–12%. Lung perfusion is performed after de-airing the circuit and connecting the pulmonary artery. Blood flow is gradually increased up to 40% of the estimated cardiac output (CO) (calculated as CO = 3*body surface area) while monitoring PA pressure (PAP). Gas flow at the circuit oxygenator is set at 5 Lt/min of a gas mix of air and 5-8% CO2. The temperature of the perfusate is gradually increased from 25 °C to a left atrium target temperature of 37 °C over approximately 30 min. Once the lung outflow temperature exceeds 32 °C, MV is started and progressively increased with a target tidal volume of 7 ml/kg of donor ideal weight, with PEEP of 5 cmH2O, respiratory rate of 7 bpm, and a FiO2 of 0.4. RMs are performed by inflating the lungs at 25 cmH2O airway pressure. An evaluation is performed every hour during EVLP by setting the ventilator at 100% of FiO2 at 1 and switching the gas flow to the circuit oxygenator from Air/CO2 to N2/CO2. Lung perfusion (perfusate flow, temperature, and PAP) and ventilation (tidal volume, plateau airway pressure, respiratory rate, PEEP, and FiO2) parameters are recorded every hour. Pulmonary vascular resistance and static lung compliance are calculated according to standard formulas. Gas exchange analysis is performed on samples drawn from the PA and left atrium.

Whenever preservation time is expected to be prolonged (≥6 hours) due to logistical problems, the Organ Care System (OCSTM, Transmedics) is usually applied.

**Lung Transplantation Surgical Technique**

The surgical approach for bilateral lung transplantation consists of two separate anterolateral thoracotomies. A bilateral trans-sternal anterior thoracotomy (clamshell incision) is used for providing additional exposure when a relatively small chest cavity makes hilar isolation difficult or if central extra-corporeal support is instituted. After lysis of pleural and mediastinal adhesions, vascular structures and bronchi are isolated. The less perfused lung (as per pulmonary perfusion study) is then disconnected from mechanical ventilation and allowed to deflate. The pulmonary artery is cross-clamped, and the first cross-clamping 10-minutes test is performed: the surgical procedure is halted, hemodynamic are strictly monitored, and blood gas analyses are obtained every 2 minutes. Whenever hemodynamic and gas exchange stability is observed, first lung implantation is performed. Close attention is paid to continuous topical cooling of the donor lung during implantation; the donor lung is placed in a bed of ice slush into the thoracic cavity. First, the bronchial anastomosis is carried out with absorbable suture materials in two continuous sutures. Pulmonary artery and atrium anastomoses performed with non-absorbable suture materials in two continuous sutures. At the time of lung reperfusion, the graft is thoroughly de-aired before vascular unclamping to avoid systemic air emboli and is then slowly re-perfused along a 10-minute period. After being connected to a separate mechanical ventilator the lung graft is progressively re-ventilated. The graft is initially ventilated in pressure control mode with FiO2 21%, PEEP of 10 cmH2O, RR of 4 bpm and plateau pressure of 25 cmH2O. A recruitment manoeuvre is applied to obtain complete lung inflation. Ventilation and oxygenation of the graft are gradually increased to allow contralateral lung separation from ventilation. Particular attention is paid in limiting 1) FiO2 (i.e., < 50%); 2) driving pressures (i.e., < 15 cmH2O) and 3) de-recruitment (i.e., PEEP > 10 cmH2O) of the implanted graft. Then, contralateral native lung ventilation is interrupted, and the second pulmonary artery cross-clamping test is performed. Pneumonectomy of the second native lung and implantation of the second graft follows the procedure described above. After accurate haemostasis, the pleural spaces are drained with two drains (no. 28 and 32French) in each pleural space. The sternum is sutured using sternal wires. The ribs are re-approached with heavy interrupted figure-of-eight multifilament absorbable suture. The pectoralis muscle, fascia and the subcutaneous tissue are approximated with monofilament absorbable suture. Skin is sutured with staples.

**ECMO indication and pulmonary artery cross-clamping test**

To assess hemodynamic and gas exchange tolerance to each single lung pneumonectomy in a controlled and reversible fashion, once hilar structures have been carefully exposed, a pulmonary artery cross-clamping test is performed. Once the anaesthesiologist has optimized hemodynamic and ventilation management, hemodynamic and gas exchange are monitored along a 10-minute period. Whenever at least one of the following conditions occurs, the cardiac surgeon is requested to implement central veno-arterial ECMO in a semi-elective condition:

1) pulmonary hypertension (i.e., systolic pulmonary artery pressure > 80 mmHg or an increase in PAPs > 50 mmHg associated with systemic hypotension (i.e., systolic arterial pressure < 60 mmHg) resistant to inotropic support;

2) major cardiac arrhythmias;

3) hypoxemia (i.e., PaO2 < 60 mmHg, despite increasing FiO2 up to 100% and optimizing PEEP) or respiratory acidosis (i.e., pH < 7.25 despite increasing minute ventilation).

During the second pulmonary-artery cross-clamping (while the first implanted graft is ventilated and perfused), our policy is more protective towards hyperoxemia and ventilator-induced lung injury. Thus, during the second test, FiO2 is not increased above 50% and airways driving pressure is not increased above 15 cmH2O. Furthermore, throughout the whole surgical procedure whenever either due to surgical reasons intractable or hypoxemia/acidosis/hemodynamic failure occurs, the surgical procedure is briefly interrupted and ECMO implemented.

After providing unfractionated heparin (i.e., 5000 UI) and eventual further boluses to achieve an aPTT > 40 seconds, the ascending aorta and right atrium are cannulated. Blood is drained via a centrifugal pump directly to a polypropylene membrane lung where blood is oxygenated, decarboxylated, warmed and then directed to the central venous circulation. Initially, blood flow is set to achieve around 50% of the patient’s cardiac output, gas flow to maintain normocapnia and fraction of oxygen in the sweep gas flow to maintain SpO2 > 95%. The extracorporeal circuit setting is dynamically modified during the procedure, depending on the different surgical and anaesthetic requirements; mean arterial pressure is maintained > 60 mmHg by increases in extracorporeal blood flow, but complete blood drainage and emptying of the heart is avoided, and the opening of the aortic valve is always guaranteed. No predefined standard management of blood components is applied, but patient-tailored transfusion management is carried out following blood gas analyses and point of care (POC) PT/aPTT tests, as well as thromboelastography, as per national guidelines.

**Recipient care after transplantation**

After transferral to the Intensive Care Unit (ICU), hemodynamic assessment is performed, thereafter tissue perfusion and cardiac function is optimized. In patients requiring post-operative VV-ECMO, intrapulmonary shunt and respiratory system compliance are obtained every 4 hours: once acceptable values are obtained, a “gas-off” trial is performed to evaluate graft function in absence of ECMO support. Whenever the interruption of extracorporeal support is tolerated, ECMO is withdrawn. Weaning from mechanical ventilation is performed by early transition to assisted mechanical ventilation and progressive weaning of pressure support and PEEP. Extubation is performed once a spontaneous breathing trial is tolerated without respiratory and hemodynamic derangements [Ouellette DR]. Non-invasive respiratory support by either C-PAP or high flow nasal cannula is frequently adopted in the early phases after invasive mechanical ventilation interruption. Physical therapy sessions are performed since post-operative day one.

At our Institution, standard immunosuppression consists of prednisone, tacrolimus, azathioprine, without specific induction therapy. Episodes of rejection are treated with intravenous methylprednisolone at a dose of 1 g/d for 3 days, followed by steroid taper. As for perioperative antibiotics, a third-generation cephalosporin or target therapy in case of specific colonization are administrated. Prophylaxis against Pneumocystis carinii (Bactrim) is standard, whereas cytomegalovirus (CMV) prophylaxis is determined by preoperative serologic status of both donor and recipient and postoperative monitoring (Ganciclovir/Valganciclovir). Patients undergo an initial routine bronchoscopic surveillance before hospital discharge. Routine surveillance bronchoscopic examinations is planned for 4 to 8 weeks postoperatively and then every 3 months during the first postoperative year. Results of pulmonary function test, radiographic findings and clinical symptoms determine the need for additional bronchoscopic evaluations. Pulmonary trans-bronchial biopsies (TBB) are scheduled at 3, 6, 12 months after transplantation.

Immunosuppression comprises methylprednisolone 1000 mg and tacrolimus or basiliximab pending the patient renal function.

**Table S1. Pulmonary function tests result at 3, 6, and 12 months after transplantation in the DCD and DBD group**

| Mean FEV1 % (sd) | | | |
| --- | --- | --- | --- |
|  | 3 months | 6 months | 12 months |
| DBD | 77. 0 (17.6) | 83.5 (17.7) | 86.0 (19.7) |
| DCD | 76.3 (21.2) | 78.5 (23.2) | 81.7 (19.5) |
|  |  |  |  |
|  | Point estimate | Standard error | p-value |
| Intercept | 77.0 | 1.77 | <0.001* |
| Time 6 months | 6.5 | 1.0 | <0.001* |
| Time 12 months | 9.0 | 1.5 | <0.001* |
| DCD Group | -0.74 | 6.34 | 0.907 |
| Time 6 months: DCD Group | -4.3 | 2.2 | 0.046* |
| Time 12 months: DCD Group | -3.5 | 2.8 | 0.208 |
| Mean FVC % (sd) | | | |
|  | 3 months | 6 months | 12 months |
| DBD | 76.0 (16.1) | 83.4 (13.7) | 89.1 (17.6) |
| DCD | 75.6 (24.9) | 81.8 (23.0) | 87.4 (22.1) |
|  |  |  |  |
|  | Point estimate | Standard error | p-value |
| Intercept | 76.0 | 1.6 | <0.001* |
| Time 6 months | 7.5 | 0.95 | <0.001* |
| Time 12 months | 13.2 | 1.3 | <0.001* |
| DCD Group | -0.3 | 7.3 | 0.970 |
| Time 6 months: DCD Group | -1.3 | 2.6 | 0.600 |
| Time 12 months: DCD Group | -1.4 | 2.0 | 0.460 |
| Mean Tiffeneau (sd) | | | |
|  | 3 months | 6 months | 12 months |
| DBD | 1.02 (0.12) | 1.00 (0.16) | 0.97 (0.13) |
| DCD | 1.03 (0.13) | 0.97 (0.17) | 0.95 (0.16) |
|  |  |  |  |
|  | Point estimate | Standard error | p-value |
| Intercept | 1.02 | 0.01 | <0.001* |
| Time 6 months | -0.01 | 0.01 | 0.170 |
| Time 12 months | -0.05 | 0.01 | <0.001* |
| DCD Group | -0.01 | 0.04 | 0.082 |
| Time 6 months: DCD Group | -0.05 | 0.03 | 0.160 |
| Time 12 months: DCD Group | -0.03 | 0.03 | 0.430 |

Respiratory function parameters were calculated in 11 and 97 patients from the DCD and DBD Group respectively. Sd: standard deviation; FEV1:

**Table S2. Pulmonary function tests results adjusted**

| FEV1 | | | |
| --- | --- | --- | --- |
|  | Point estimate | Standard error | p-value |
| Intercept | 105.2 | 16.6 | <0.001* |
| Time 6 months | 6.6 | 1.0 | <0.001* |
| Time 12 months | 8.9 | 1.5 | <0.001* |
| DCD Group | 3.11 | 5.8 | 0.590 |
| Sex mismatch | 0.01 | 4.0 | 0.998 |
| Incision: Clamshell | -8.9 | 3.1 | 0.004* |
| Disease: Chronic obstructive pulmonary disease | -3.8 | 13.5 | 0.776 |
| Disease: Cystic fibrosis | -10.4 | 11.2 | 0.349 |
| Disease: Interstitial lung disease | -10.6 | 11.6 | 0.361 |
| Disease: Pulmonary vascular disease | -41.4 | 11.9 | <0.001* |
| Disease: Other | -13.1 | 13.9 | 0.346 |
| LAS | -0.14 | 0.13 | 0.265 |
| Grade 3 PGD: Yes | -12.5 | 3.7 | <0.001* |
| TIT 1^st^ lung | -0.06 | 0.03 | 0.096 |
| TIT 2^nd^ lung | 0.05 | 0.03 | 0.144 |
| WIT 1^st^ lung | -0.06 | 0.1 | 0.226 |
| WIT 2^nd^ lung | 0.03 | 0.1 | 0.680 |
| Donor smoking history: Yes | 6.5 | 5.0 | 0.199 |
| Donor smoking history: No | 0.05 | 4.96 | 0.992 |
| Donor age | -0.14 | 0.11 | 0.205 |
| Airway complications | -4.1 | 5.04 | 0.218 |
| Time 6 months: DCD Group | -4.4 | 2.2 | 0.042* |
| Time 12 months: DCD Group | -3.5 | 2.8 | 0.216 |
| FVC | | | |
|  | Point estimate | Standard error | p-value |
| Intercept | 81.7 | 18.1 | <0.001* |
| Time 6 months | 7.6 | 1.0 | <0.001* |
| Time 12 months | 13.1 | 1.3 | <0.001* |
| DCD Group | 1.7 | 5.9 | 0.773 |
| Sex mismatch | 4.2 | 4.0 | 0.291 |
| Incision: Clamshell | -8.4 | 2.7 | 0.002* |
| Disease: Chronic obstructive pulmonary disease | 3.2 | 15.6 | 0.839 |
| Disease: Cystic fibrosis | 0.85 | 14.1 | 0.952 |
| Disease: Interstitial lung disease | -3.4 | 14.5 | 0.816 |
| Disease: Pulmonary vascular disease | -16.4 | 14.4 | 0.255 |
| Disease: Other | -8.5 | 16.2 | 0.602 |
| LAS | -0.07 | 0.03 | 0.551 |
| Grade 3 PGD: Yes | -8.6 | 3.5 | 0.015* |
| TIT 1^st^ lung | -0.07 | 0.03 | 0.048* |
| TIT 2^nd^ lung | 0.05 | 0.03 | 0.136 |
| WIT 1^st^ lung | -0.14 | 0.1 | 0.147 |
| WIT 2^nd^ lung | 0.02 | 0.1 | 0.794 |
| Donor smoking history: Yes | 7.9 | 5.6 | 0.158 |
| Donor smoking history: No | 1.7 | 5.3 | 0.752 |
| Donor age | 0.15 | 0.1 | 0.142 |
| Airway complications | 7.9 | 5.9 | 0.183 |
| Time 6 months: DCD Group | -1.4 | 2.6 | 0.590 |
| Time 12 months: DCD Group | -1.4 | 1.97 | 0.481 |
| Tiffeneau | | | |
|  | Point estimate | Standard error | p-value |
| Intercept | 1.27 | 0.099 | <0.001* |
| Time 6 months | -0.01 | 0.01 | 0.155 |
| Time 12 months | -0.05 | 0.01 | <0.001* |
| DCD Group | 0.01 | 0.03 | 0.582 |
| Sex mismatch | -0.05 | 0.26 | 0.075 |
| Incision: Clamshell | -0.0005 | 0.020 | 0.981 |
| Disease: Chronic obstructive pulmonary disease | -0.08 | 0.063 | 0.209 |
| Disease: Cystic fibrosis | -0.13 | 0.057 | 0.026* |
| Disease: Interstitial lung disease | -0.08 | 0.059 | 0.149 |
| Disease: Pulmonary vascular disease | -0.37 | 0.062 | <0.001* |
| Disease: Other | -0.06 | 0.084 | 0.479 |
| LAS | -0.001 | 0.001 | 0.231 |
| Grade 3 PGD: Yes | -0.05 | 0.024 | 0.036* |
| TIT 1^st^ lung | 0.00003 | 0.0002 | 0.882 |
| TIT 2^nd^ lung | 0.0001 | 0.002 | 0.619 |
| WIT 1^st^ lung | 0.0001 | 0.0005 | 0.804 |
| WIT 2^nd^ lung | 0.00002 | 0.0007 | 0.978 |
| Donor smoking history: Yes | -0.01 | 0.040 | 0.736 |
| Donor smoking history: No | -0.2 | 0.039 | 0.542 |

**Table S3. Pulmonary function tests result at 3, 6, and 12 months after transplantation in the DCD and EVLP-DBD group**

|  | Mean FEV1 % (sd) | | |
| --- | --- | --- | --- |
|  | 3 months | 6 months | 12 months |
| DBD-EVLP | 74.8 (17.5) | 79.1 (13.8) | 80.4 (14.5) |
| DCD | 76.3 (21.2) | 78.5 (23.2) | 81.7 (19.5) |
|  |  |  |  |
|  | Point estimate | Standard error | p-value |
| Intercept | 74.8 | 4.5 | <0.001* |
| Time 6 months | 4.3 | 2.8 | 0.125 |
| Time 12 months | 5.5 | 5.5 | 0.314 |
| DCD Group | 1.41 | 7.6 | 0.852 |
| Time 6 months: DCD Group | -2.1 | 3.4 | 0.534 |
| Time 12 months: DCD Group | -0.05 | 5.9 | 0.994 |
|  | Mean FVC % (sd) | | |
|  | 3 months | 6 months | 12 months |
| DBD-EVLP | 75.7 (16.2) | 80.0 (13.4) | 85.6 (12.9) |
| DCD | 75.6 (24.9) | 81.8 (23.0) | 87.4 (22.1) |
|  |  |  |  |
|  | Point estimate | Standard error | p-value |
| Intercept | 75.7 | 4.2 | <0.001* |
| Time 6 months | 4.3 | 2.6 | 0.098 |
| Time 12 months | 9.9 | 4.1 | 0.016* |
| DCD Group | -0.1 | 8.3 | 0.992 |
| Time 6 months: DCD Group | 1.9 | 3.6 | 0.593 |
| Time 12 months: DCD Group | 1.9 | 4.4 | 0.669 |
|  | Mean Tiffeneau (sd) | | |
|  | 3 months | 6 months | 12 months |
| DBD | 0.99 (0.16) | 0.99 (0.13) | 0.95 (0.16) |
| DCD | 1.03 (0.13) | 0.97 (0.17) | 0.95 (0.16) |
|  |  |  |  |
|  | Point estimate | Standard error | p-value |
| Intercept | 0.99 | 0.04 | <0.001* |
| Time 6 months | 0.002 | 0.22 | 0.920 |
| Time 12 months | -0.05 | 0.03 | 0.130 |
| DCD Group | 0.03 | 0.06 | 0.590 |
| Time 6 months: DCD Group | -0.06 | 0.04 | 0.110 |
| Time 12 months: DCD Group | -0.03 | 0.05 | 0.560 |

Respiratory function parameters were calculated in 11 and 14 patients from the DCD and EVLP-DBD Group respectively. Sd: standard deviation; EVLP: ex-vivo lung perfusion; FEV1: Forced Expiratory Volume in the first second; FVC: Forced Vital Capacity.
